# HLA-E restricted cytomegalovirus UL40 peptide polymorphism may represent a risk factor following congenital infection

**DOI:** 10.1186/s12864-022-08689-0

**Published:** 2022-06-20

**Authors:** David Tarragó, Irene González, Maria Francisca González-Escribano

**Affiliations:** 1 National Center for Microbiology, Instituto de Salud Carlos III, Majadahonda- Pozuelo km 2, 28220 Majadahonda Madrid, Spain; 2 CIBER Epidemiology and Public Health (CIBERESP), Madrid, Spain; 3 Department of Immunology, Virgen del Rocío Universitary Hospital (IBiS, CSIC, US), 41013 Seville, Spain

**Keywords:** Congenital CMV, UL40 peptide, Immunomodulatory gene, UL40 variability, HLA-E

## Abstract

**Background:**

Congenital cytomegalovirus immunopathogenesis is largely unknown and multifactorial due to the complex interactions between viral, maternal, placental, and child factors. Polymorphisms in the HLA-E binding UL40_15-23_ peptide mimics HLA-E complexed peptides from certain HLA-A, -B, -C and -G alleles, which regulate the cellular immune response driven by natural killer-cells (NK) and CD8 + T cells. The aim of this study was to compare UL40_15-23_ peptides distribution in congenital CMV and the counterpart HLA Class I peptides in a healthy cohort to investigate risk factors and markers for cCMV disease. In this 10-year retrospective study, the *UL40 gene* was directly sequenced from 242 clinical samples from 199 cases of congenital CMV (166 children and 33 pregnant or breast feeding women). Distribution of HLA-E binding UL40_15-23_ peptides was analyzed and compared to those of HLA Class I observed in a cohort of 444 healthy individuals.

**Results:**

Nineteen different HLA-E binding UL40_15-23_ peptides were found. Three of them (VMAPRTLIL, VMAPRTLLL, VMAPRTLVL) were found in 88.3% of UL40 and 100% of HLA Class I of healthy individuals. In contrast, 15 of them (10.7%) were not found in HLA Class I. The VMAPRTLFL peptide was found in 1% of UL40 and all HLA-G alleles. Significant differences in peptide (VMAPRTLIL, VMAPRTLLL, VMAPRTLVL, other UL40_15-23_ peptides, other HLA Class I peptides) distribution between UL40_15-23_ from congenital CMV and HLA-A, -B, -C and –G from healthy individuals were found.

**Conclusions:**

Our findings suggest that a mismatch between UL40_15-23_ peptides and HLA Class I peptides between children and mothers might play a role in congenital CMV disease, and it may account for differences in outcome, morbidity and sequelae.

**Supplementary Information:**

The online version contains supplementary material available at 10.1186/s12864-022-08689-0.

## Background

Congenital cytomegalovirus (cCMV) is the most common congenital infection in the world and a leading cause of sensorineural hearing loss, mental retardation, microcephaly, development delay, seizure disorders, and cerebral palsy [1]. Approximately 10% of congenitally infected infants have symptoms at birth, and sequelae occur in 40– 58% of them and in 13.5% of asymptomatic infants [2]. The causes for these differences in outcome remain unknown and are probably multifactorial, and may include different CMV pathogenicity in the context of the genetic backgrounds of the fetus and mother and other known factors such as when primary infection occurs in the first trimester of pregnancy [3]. Once CMV infects the placenta, local damage and inflammation lead to placental dysfunction, which in turn affects fetal development [4].

The activity of NK cells is controlled by the binding of CD94-NKG2 receptors to HLA-E complexed peptides from HLA-A, -B, -C and -G alleles expressed on the cell surface [5] and polymorphic UL40 peptides from CMV shape the adaptive NKG2C NK cell populations [6]. HLA-C, HLA-E, and HLA-G are the only HLA molecules expressed in the placenta [7] and HLA-C and HLA-E prevent maternal NK cell-mediated cytotoxicity through binding killer-cell immunoglobulin-like receptors (KIRs) expressed on decidua killer-cells. Some combinations of maternal KIRs and fetal HLA-C can lead to pregnancy complications [8]. HLA-E also presents these HLA peptides to CD8^+^ T cells to trigger specific cellular immune responses [9, 10]. Infection with CMV can trigger a CD8^+^ T cell response restricted by HLA-E and are specific for a peptide from UL40 (VMAPRTLIL) [11], which is characterized by biased *TRBV14 gene* usage [12]. Other viral and host factors (separate to the HLA-E binding peptides) could influence the outcome: The specific function of the rest of the *UL40 gene* is unknown, while the HLA-E binding peptide also alters expression of UL18 [13], which in turn binds leukocyte Ig-like receptor 1 (LIR1) with high affinity. LIR1 also binds HLA Class I molecules. More recently, heterogeneity in the UL18/LIR1 interaction has been associated with altered control of CMV in kidney transplant recipients [14].

We hypothesize that polymorphism in this viral ligand in the context of a particular HLA-A, -B, -C and -G allele of children and mothers may influence the immune response and consequently different cCMV infection outcomes may be expected.

The aim of this study was to gain more insight into cCMV pathogenesis and its clinical consequences by describing the variability and distribution of UL40_15- 23_ CMV peptide in a large cohort of children with congenital infection and mothers with an affected fetus or newborns. Moreover, it is relevant to establish whether the distribution of different peptides is the same in the healthy population as in the CMV strains that are responsible of congenital infection Therefore, in order to determine whether polymorphism in the UL40_15-23_ peptide represent a risk factor, we compared its occurrence to variability and distribution of these peptides derived from HLA-A, -B, -C and -G alleles in a healthy population.

## Results

### Distribution of UL40_15-23_ peptides in congenital CMV infection

Two hundred forty-two *UL40 gene* sequences were analyzed. Different UL40 polymorphisms were found, particularly in the region encoding the main potential HLA-E binding peptide. Once the HLA-E binding fragment was translated and selected, 19 different UL40_15-23_ peptides (9-mers) with different binding properties and distribution amongst patients were found (Table 1). The 8^th^ amino acid position was observed to be the most variable (22^nd^ position in UL40). VMAPRTLLL, VMAPRTLVL and VMAPRTLIL peptides account for 176/199 (88.4%) of all UL40_15-23_ peptides analyzed and they matched with most HLA-A, -B, or -C alleles of HLA IPD- IMGT/HLA Database (Table 1 Supplementary). The UL40_15-23_ peptide VMAPRTLFL was found in only two patients and matched all HLA-G alleles. In addition to the three most predominant UL40_15-23_ peptides, another four UL40_15-23_ peptides matched with a specific HLA Class I allele, while the remaining 11 did not match any allele, as shown in detail in Table 1 Supplementary.

**Table 1:** UL40_15-23_ peptides found in CMV strains from 199 patients and HLA-E predicted binders:

9-mer UL40_15-23_ (accession number)	(n) % patients	% OPT	Cleavage model predicted^a^	Binding Score^b^
LMAPRTLLL (9)	(1) 0.5	97.54	Yes	277.0
VIAPRTLIL (17)	(1) 0.5	90.85	yes	258.0
VLAPRTLLL (7)	(1) 0.5	95.42	yes	271.0
VMAPRILIL (4)	(2) 1	80.99	yes	230.0
VMAPRILVL (11)	(4) 2	80.28	yes	228.0
VMAPRSLIL (15)	(1) 0.5	87.68	yes	249.0
VMAPRSLLL (6)	(2) 1	89.44	yes	254.0
VMAPRTLFL(12)	(2) 1	91.90	yes	261.0
VMAPRTLIL (3)	(120) 60.3	98.24	yes	279.0
VMAPRTLIM (5)	(1) 0.5	94.01	No	267.0
VMAPRTLIV (18)	(1) 0.5	95.77	yes	272.0
VMAPRTLLL (1)	(20) 10	100	yes	284.0
VMAPRTLVL (2)	(36) 18	97.54	yes	277.0
VMDPRTLIL (13)	(1) 0.5	88.03	yes	250.0
VMGPRTLLL (14)	(1) 0.5	91.55	yes	260.0
VMTPRTLIL (19)	(1) 0.5	90.85	yes	258.0
VMTPRTLLL(10)	(1) 0.5	92.61	yes	263.0
VMTPRTLVL (8)	(2) 1	90.14	yes	256.0
VMVPRTLVL (16)	(1) 0.5	88.03	yes	250.0

% OPT is the percentile score of the predicted peptide relative to that of the *consensus*. The *consensus *is the sequence that yields the maximum score, namely *optimal score (OPT) *with the selected profile. ^a^Proteasomal cleavage predictions was carried out using three optional models obtained applying statistical language models to a set of knwon epitopes restricted by human MHCI molecules. * The binding potential (score) of any peptide sequence (query) to a given MHCI is obtained by aligning the relevant PSSM with the protein segments, and adding up the profile scores that match the residue type and position in the profile. ^b^Binding Score according to maximum score of 284 obtained with VMAPRTLLL. GenBank accession numbers: (1) OM397419, (2) OM397420, (3) OM397421, (4) OM397422, (5) OM397423, (6) OM397424, (7) OM397425, (8) OM397426, (9) OM397427, (10) OM397428, (11) OM397429, (12) OM397430, (13) OM397431, (14) OM397432, (15) OM397433, (16) OM397434, (17) OM397435, (18) OM397436, (19) OM397437.

### Distribution of HLA Class I peptides in healthy population

There is only one peptide copy of CMV per patient. However, the homologous peptide (HLA) in humans may be duplicated in each HLA Class I. Therefore, it may be the case that in humans there are two copies of a peptide (in HLA-C), another two copies in HLA-A and so on. In addition, the same individual has heterozygous peptides (two different HLA- C peptides, for example). We must consider this when statistically analyzing the 444 individuals. Therefore, we obtained 403 VMAPRTLIL and 76 VMAPRTLLL peptides in HLA-C that do not add up to 444. Distribution of HLA-A, B, C and G peptides in 444 healthy individuals was as follows:

HLA-A: All individuals belonged to one of these three groups: VMAPRTLLL/VMAPRTLLL 116(26%), VMAPRTLLL/VMAPRTLVL 220(49.5%), VMAPRTLVL/VMAPRTLVL 108(24.3%). HLA-C: VMAPRTLIL/VMAPRTLIL 342(77%), VMAPRTLIL/VMAPRTLLL 61(13.7%), VMAPRTLLL/VMAPRTLLL 15(3.4%), none of them 26(5.9%); VMAPRALLL or VMAPQALLL corresponding to HLA-C*07:18 and HLA-C*17 alleles, respectively). HLA-B: None. HLA-G: All were VMAPRTLFL.

Thus, not taking into account the copy number and locus, we found 354 individuals with VMAPRTLLL peptide; 328 with VMAPRTLVL peptide; 403 individuals with VMAPRTLIL peptide and 444 individuals with VMAPRTLFL peptide. Detailed data are shown in Table 2.

**Table 2:** UL40 9-mer found in cCMV versus HLA Class I of 444 healthy individuals

9-mer UL40_15-23_	N HLA-C	N HLA-A	N HLA-B	N HLA-G
LMAPRTLLL	0	0	0	0
VIAPRTLIL	0	0	0	0
VLAPRTLLL	0	0	0	0
VMAPRILIL	0	0	0	0
VMAPRILVL	0	0	0	0
VMAPRSLIL	0	0	0	0
VMAPRSLLL	0	0	0	0
VMAPRTLFL	0	0	0	444
VMAPRTLIL	403	0	0	0
VMAPRTLIM	0	0	0	0
VMAPRTLIV	0	0	0	0
VMAPRTLLL	76	336	0	0
VMAPRTLVL	0	328	0	0
VMDPRTLIL	0	0	0	0
VMGPRTLLL	0	0	0	0
VMTPRTLIL	0	0	0	0
VMTPRTLLL	0	0	0	0
VMTPRTLVL	0	0	0	0
VMVPRTLVL	0	0	0	0
-	26^a^			

^a^Nonamers not found in UL40_15-23_ (VMAPRALLL or VMAPQALLL)

### Comparison between HLA Class I peptides from healthy individuals and UL40 peptides from cCMV

**Fig. 1:**
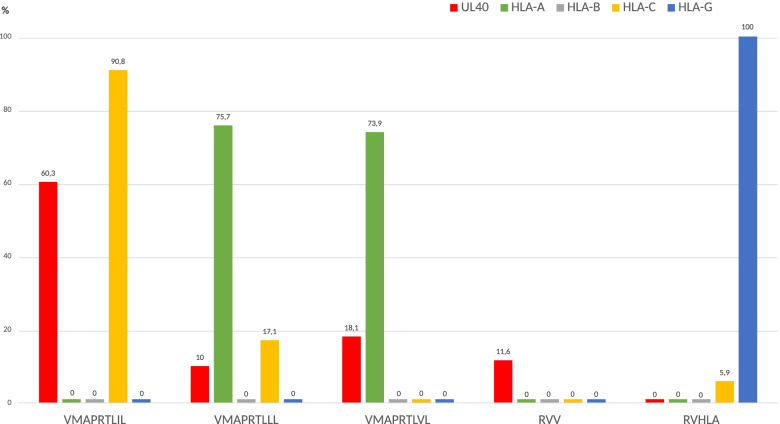
Peptide distribution comparisons between UL40_15-23_ and HLA-A, -B, -C, -G. Figure 1 foot: RVV = other UL40_15-23_ peptides; RVHLA = other HLA Class I peptides

Only four of the 19 peptides obtained in UL40_15-24_ CMV (VMAPRTLIL, VMAPRTLLL, VMAPRTLVL, and VMAPRTLFL) were also found in HLA Class I from healthy individuals (Table 2). VMAPRTLIL and VMAPRTLFL peptides were only found in HLA-C and HLA-G, respectively. A significant difference in peptide distribution between UL40_15-23_ and HLA-A, -B, -C and -G was found (Fig. 1). Interestingly, significant differences in VMAPRTLIL and VMAPRTLLL peptides between UL40_15-23_ and HLA-C were not as broad as in the other HLA Class I loci (*p* = 1.561797e-18 and *p* = 2.260558e-02, respectively). Comparisons and their significance in peptide distribution between UL40_15-23_ and HLA-A, -B, -C, -G were as follows: (VMAPRTLIL (*p* = 8.637e-77, *p* = 8.637e-77, *p* = 1.561e-18, *p* = 8.637e-77); VMAPRTLLL (*p* = 1.578e-57, *p* = 4.350e-11, *p* = 2.260e-02, *p* = 4.350e-11); VMAPRTLVL (*p* = 1.581e-40, *p* = 4.283e-20, *p* = 4.283e-20, *p* = 4.283e-20); other UL40_15-23_ peptides ((*p* = 7.646e-13, *p* = 7.646e-13, *p* = 7.646e-13, *p* = 1.104e-132); other HLA Class I peptides (*p* = 1, *p* = 1, *p* = 3.953e-4, *p* = 1).

### Limitations of study and assumptions

Assumptions:

1. In cCMV, UL40_15-23_ peptides that are equal to the HLA peptides of the patients may be selected, therefore, they should show a distribution without significant differences with respect to the HLA Class I peptides.
2. We assume that the distribution of HLA Class I peptides in a healthy population and in congenitally CMV infected children was the same.

Limitations:

1. It would be best to compare the peptide of the virus with the HLA class I peptide of the same patient (paired data) but this is impossible to obtain in this retrospective study, so we do not have this HLA data for the patient. An approach to the above was to study these peptides among a healthy population and to analyze whether this distribution of peptides is statistically similar or different from that obtained of the viruses of the patients. In further studies, it should be interesting correlate the findings with more detailed clinical data.
2. CMV Sanger sequencing reveals the most abundant *UL40 gene* CMV variants (> 30%). However, coinfection with less frequent variants should not be ruled out.

## Discussion

The CMV UL40 signal peptide contains a 9-mer sequence that is processed in the same way as endogenous HLA-E binding peptides and, consequently when bound to HLA-E can promote CD8 T cells and NK responses. A wider range of UL40_15-23_ peptides in cCMV than in their counterpart HLA peptides from healthy donor individuals was observed: Up to 19 different UL40_15-23_ nonamers were found in clinical samples of 242 patients with cCMV infection. In contrast, three of them were present in all healthy donor individuals, although differently distributed among HLA Class I loci. These three endogenous HLA Class I peptides were found in 60.3% (VMAPRTLIL UL40_15-23_), 18% (VMAPRTLVL UL40_15-23_) and 10% (VMAPRTLLL UL40_15-23_) of cCMV strains. When we compared the distribution between peptides from HLA-A, -B, -C, -G and UL40_15-23_, significant differences were found. The importance of matching UL40_15-23_/ HLA-derived peptides has been described previously: a strong VMAPRTLIL-UL40-specific, CD8^+^ T cell response was observed in individuals who lack HLA-C alleles that encode this determinant [10, 15]. Selective pressure may drive the UL40_15-23_ peptide to be more adapted to the HLA alleles of hosts. The VMAPRTLFL UL40_15-23_ peptide was found only in two CMV strains, but it could be found in all HLA-G alleles, which is expressed only by trophoblast cells. In addition, any UL40_15-23_ peptide observed was not found in HLA-B of the cohort of healthy individuals (VMAPRTLLL is specific of a rare HLA-B*13:117 allele) and the fact that HLA-A and B are not expressed by trophoblasts suggests a main role of HLA-C derived peptides in cCMV pathogenesis. It is interesting that when we compare peptide distribution between UL40_15-23_ CMV from patients and peptides derived from HLA-C of healthy individuals a significant different distribution was found although the VMAPRTLIL peptide was the most prevalent peptide in UL40 and HLA-C.

Moreover, a study with blood donors and kidney transplant recipients demonstrated that CMV induces strong HLA-E_UL40_ CD8 T cells responses with potential allogeneic or/and autologous reactivity depending on virus strain and host HLA concordance [15]. This finding may be relevant in cCMV pathogenesis. Regarding the tolerating state of the fetus against allopeptides from the mother, different situations may be noted: CMV strains which produce UL40_15-23_ peptides that do not correspond with endogenous HLA-E binding peptides of children and their mothers may elicit robust HLA-E restricted T cells responses against viral ligands. Other conditions may be found when only mother or fetus are matched. On the contrary, CMV strains that produce UL40_15-23_ peptides, which match with allopeptides from mothers and children, may produce a tolerating state against these viral ligands. Of course, whether this is critical or not for stimulating strong allogeneic or/and autologous reactivity in cCMV requires further studies. In our study, a significant difference in UL40_15-23_ peptides from cCMV disease compared to those found in HLA Class I from healthy individuals was detected. Although many other viral and host factors are involved in cCMV, this variation suggests that it may play a role in cCMV pathogenesis. Paired CMV/HLA Class I genotyping cCMV studies are needed to validate this proposal.

The rationale for this study was to explore how HLA-E restricted cytomegalovirus UL40 peptide polymorphism are associated with risk for CMV disease following congenital infection. Our findings suggest that UL40 peptide polymorphisms are indeed associated with disease, and thus the findings reported here open new windows to explore potential biomarkers of congenital CMV disease that help clinicians in the management of pediatric patients. In this sense, characterization of UL40 nonamer and HLA- Class I nonamer in a patient (child and mother) could provide useful information regarding prognosis of the disease and it should facilitate clinical decision regarding the use of antivirals or a more extensive follow up of the patient. This new approach together with the knowledge of these mechanisms and biomarkers is linked to the principles of personalized medicine, which is undoubtedly being developed and will be developed in the future.

## Conclusions

In the present study, we characterized the profile of HLA-E binding peptides of CMV in congenital infection. Our findings suggested that a mismatch between UL40_15-23_ peptides and the HLA Class I peptides of children and mothers might play a role in congenital CMV disease, and it may account for differences in outcome, morbidity and sequelae. To our knowledge, this study represents a novelty approach and the description of the highest number of different UL40_15-23_ peptides found in CMV disease. The above findings provided unique insights for further studies on CMV/host interactions in congenital infection and could serve as the basis for the development of molecular markers of disease.

## Methods

### Patients and specimens

Residual clinical specimens submitted for virological diagnosis of cCMV infection from 42 Spanish hospitals from 2009 to 2019 were analyzed. The use of these samples was approved by the Ethics Committee of the “Instituto de Salud Carlos III” (CEI PI 41_2016-v2). A total of 242 CMV PCR-positive clinical samples from 166 children younger than 6 years old diagnosed with cCMV infection (207 clinical samples) and 33 pregnant or breast feeding women (35 clinical samples) were available for the study. Children´s age ranged from less than 1-day-old to 6 years old with a median of 272 days old. 38% of them were females. Pregnant or breast-feeding women were a median of 31 years old. There was no reported epidemiological relationship among children of either pregnancy or breast-feeding women, except for a case of three newborn triplets. In this retrospective study, cCMV infection was diagnosed by neonatal symptoms: mainly, the infant being small for their gestational age (SGA, growth restriction < 10th centile), but also by abnormal clinical examination: hydrops, petechia or purpura, hepatosplenomegaly, microcephaly, hypotonia, sucking difficulties, lethargy, seizures; abnormal laboratory parameters: platelet count < 100 000/mm^3^, haemoglobin level < 11 g/dl, alanine aminotransferase level > 80 IU/L, conjugated bilirubin plasma level > 20 µmol/L and > 10% of total bilirubin; or severe abnormality on cerebral imaging (ultrasound and/or cranial computed tomographic scan): multiple intracranial calcifications, periventricular hyper echogenicity, severe ventriculomegaly (> 15 mm); or abnormal funduscopic examination or abnormal audiology assessment. In pregnant women, by pathological ultra sound images of fetus, and in breast feeding women, by laboratory confirmed prenatal primary CMV infection and some neonatal symptoms described above. The 242 clinical samples were distributed as follows: 157 urine, 24 amniotic fluid, 23 blood spots on paper, 14 blood, seven breast milk, four placenta, three sera, two CSF, two spleen biopsies, one bowel biopsy, one liver biopsy, one brain biopsy, one bone marrow biopsy, one nasopharyngeal wash and one nasopharyngeal aspirate.

### Cohort of healthy individuals

A total of 444 healthy individuals were included in the study. They were unrelated Spanish European adults (50% males) from HLA genotyped healthy controls previously used in another study [16]. Detailed genotype data used in this study were provided by MF González-Escribano, Head of Department of Immunology of Virgen del Rocio Universitary Hospital, Seville, Spain.

### DNA extraction, PCR and sequencing

CMV positive clinical samples were directly sequenced for *UL40 gene* using a nested PCR assay previously described by Garrigue I. et al. [17] with minor modifications. Briefly, DNA was extracted using QIAsymphony system and QIAamp DSP DNA Midi Kit (Qiagen) from 400μL of clinical sample. A CMV *UL40 gene* fragment (666 bp) was amplified using the Platinum SuperFi DNA Polymerase reaction kit (Invitrogen). PCR products were processed for Sanger dideoxy sequencing with BigDye v. 3.1 (Applied Biosystems) in an ABI PRISM 3100 sequencer (Applied Biosystems, California, USA) at the Genomic Department of National Center for Microbiology. DNA sequence analysis was carried out using the Lasergene SeqMan software. Alignments of raw sequence data were performed against expected amplicons. Afterwards, all sequences were imported, aligned and translated by MEGA 7 software using the *UL40 gene* sequence from Human Herpesvirus 5 (Merlin strain) as reference sequence (NCBI Reference Sequence: NC_006273.2).

### Peptide HLA Class I allele search and prediction of MHC- restricted ligands

Peptide HLA class-I allele search was performed using the Immune Polymorphism Database (IPD-IMGT/HLA Database) and the sequence alignment tool from EMBL-EBI: https://www.ebi.ac.uk/ipd/imgt/hla/align.html. CMV UL40 peptide binding were predicted using RANKPEP (http://imed.med.ucm.es/Tools/rankpep.html) [18]. RANKPEP uses Position Specific Scoring Matrices or profiles from a set of aligned peptides known to bind to HLA-E as the predictor of MHC-peptide binding. The OPT percentage is the percentile score of the predicted peptide relative to that of the *consensus*. The *consensus* is the sequence that yields the maximum score, namely *optimal score* (OPT), with the selected profile.

### Statistical analysis of data

A descriptive analysis of the variables was performed. To compare peptide distributions Fisher exact test were used. *P*-values were adjusted for multiple comparisons using Benjamini–Hochberg method. Statistical significance was defined as *p* < 0.05 for all tests. All statistical analyses were performed using R software (R 3.6.2;R foundation for Statistical Computing, Vienna, Austria).

## Supplementary Information

**Additional file 1: Table 1.** Supplementary data. HLA-A, -B, -C and -G alleles from IPD-IMGT/HLA Database with 100% identity with 9-mer UL40_1524_ CMV found in this study.

## Data Availability

The datasets generated and analysed during the current study are available in the Genbank (NCBI) repository (https://www.ncbi.nlm.nih.gov/Genbank) Project PRJEB50267 with accession numbers GenBank OM397419 to OM397437.

## Abbreviations

CMV: Cytomegalovirus
HLA: Human leukocyte antigen
cCMV: Congenital cytomegalovirus
NK: Natural killer-cells
KIR1: Leukocyte Ig-like receptor-1
CEI: Comité de Ética e Investigación del Instituto de Salud Carlos III
