# Supplementary material for: HLA-E restricted cytomegalovirus UL40 peptide polymorphism may represent a risk factor following congenital infection
Source: BMC Genomics. 2022 Jun 20;23:455. doi: 10.1186/s12864-022-08689-0 (PMC9208114; doi:10.1186/s12864-022-08689-0)
Supplement: Supplementary file 1 — Additional file 1: Table 1. Supplementary data. HLA-A, -B, -C and -G alleles from IPD-IMGT/HLA Database with 100% identity with 9-mer UL401524 CMV found in this study. [file 12864_2022_8689_MOESM1_ESM.docx]

Table 1 supplementary data. HLA-A, -B, -C and -G alleles from IPD-IMGT/HLA Database with 100% identity with 9-mer UL40_1524_ CMV found in this study.

| 9-mer UL40_1524_ | HLA-A* | HLA-B* | HLA-C* | HLA-G* |
| --- | --- | --- | --- | --- |
| LMAPRTLLL | HLA-A*03:349; HLA-A*25:60 | NOT FOUND | NOT FOUND | NOT FOUND |
| VIAPRTLIL | NOT FOUND | NOT FOUND | HLA-C*08:178 | NOT FOUND |
| VLAPRTLLL | NOT FOUND | NOT FOUND | NOT FOUND | NOT FOUND |
| VMAPRILIL | NOT FOUND | NOT FOUND | NOT FOUND | NOT FOUND |
| VMAPRILVL | NOT FOUND | NOT FOUND | NOT FOUND | NOT FOUND |
| VMAPRSLIL | NOT FOUND | NOT FOUND | HLA-C*03:358 | NOT FOUND |
| VMAPRSLLL | NOT FOUND | NOT FOUND | NOT FOUND | NOT FOUND |
| VMAPRTLFL | NOT FOUND | NOT FOUND | NOT FOUND | HLA-G*01:01 a 01:22 (All of them)) |
| VMAPRTLIL | HLA-A*02:830 | NOT FOUND | From HLA-C*01:02:01 to HLA-C*01:177; from HLA-C*03:02:01 to HLA-C*06:263N except: HLA-C*03:227, HLA-C*03:358, HLA-C*03:417, HLA-C*06:17, HLA-C*06:236, HLA-C*06:239, HLA-C*06:244, HLA-C*06:261; from HLA-C*08:01 to HLA-C*14:109; HLA-C*15:43; from HLA-C*16:01 to HLA-C*16:152 except HLA-C*16:121 | NOT FOUND |
| VMAPRTLIM | NOT FOUND | NOT FOUND | NOT FOUND | NOT FOUND |
| VMAPRTLIV | NOT FOUND | NOT FOUND | NOT FOUND | NOT FOUND |
| VMAPRTLLL | From HLA-A*01:01 to HLA-A*01:305 except: HLA-A*01:234, HLA-A*01:237, HLA-A*01:295, HLA-A*01:296; from HLA-A*03:01 to HLA-A*11:332 except: HLA-A*03:242, HLA-A*03:312, HLA-A*11:320; from HLA-A*29:01 to HLA-A*33:183 except: HLA-A*29:95, HLA-A*30:144, HLA-A*30:147, HLA-A*31:143, HLA-A*31:153, HLA-A*32:114, HLA-A*33:171, HLA-A*33:172; from HLA-A*36:01 to HLA-A*36:08; from HLA-A*74:01 to HLA-A*74:35 | HLA-B*13:117 | From HLA-C*02:02:01 to HLA-C*02:169N except HLA-C*02:163; HLA-C*04:226, HLA-C*04:167, HLA-C*04:243; from HLA-C*15:02:01 to HLA-C*15:02:02:02; from HLA-C*15:02:25 to HLA-C*15:194; HLA-C*16:121 | NOT FOUND |
| VMAPRTLVL | From HLA-A*02:01 to HLA-A*02:837 except HLA-A*02:759, HLA-A*02:761, HLA-A*02:765, HLA-A*02:776, HLA-A*02:819, HLA-A*02:830; including HLA-A*03:242, HLA-A*03:312; from HLA-A*23:01 to HLA-A*26:184 except: HLA-A*24:352, HLA-A*25:60, HLA-A*26:183; including HLA-A*34:01, HLA-A*34:06, HLA-A*34.08, HLA-A*34:20, HLA-A*34:21, HLA-A*34:22, HLA-A*43:01. from HLA-A*66:01 to HLA-A*66:03, HLA-A*66:13, HLA-A*66:17, HLA-A*66:24. From HLA-A*66:27N to HLA-A*69:05 except HLA-A*68:198. | NOT FOUND | HLA-C*06:236 | NOT FOUND |
| VMDPRTLIL | NOT FOUND | NOT FOUND | NOT FOUND | NOT FOUND |
| VMGPRTLLL | NOT FOUND | NOT FOUND | NOT FOUND | NOT FOUND |
| VMTPRTLIL | NOT FOUND | NOT FOUND | NOT FOUND | NOT FOUND |
| VMTPRTLLL | NOT FOUND | NOT FOUND | NOT FOUND | NOT FOUND |
| VMTPRTLVL | NOT FOUND | NOT FOUND | NOT FOUND | NOT FOUND |
| VMVPRTLVL | HLA-A*02:776 | NOT FOUND | NOT FOUND | NOT FOUND |
